# Wildlife Conservation at a Garden Level: The Effect of Robotic Lawn Mowers on European Hedgehogs (*Erinaceus europaeus*)

**DOI:** 10.3390/ani11051191

**Published:** 2021-04-21

**Authors:** Sophie Lund Rasmussen, Ane Elise Schrøder, Ronja Mathiesen, Jeppe Lund Nielsen, Cino Pertoldi, David W. Macdonald

**Affiliations:** 1 Wildlife Conservation Research Unit, The Recanati-Kaplan Centre, Department of Zoology, University of Oxford, Tubney House, Abingdon Road, Tubney, Abingdon OX13 5QL, UK; david.macdonald@zoo.ox.ac.uk; 2 Department of Chemistry and Bioscience, Aalborg University, Fredrik Bajers Vej 7H, DK-9220 Aalborg, Denmark; jln@bio.aau.dk (J.L.N.); cp@bio.aau.dk (C.P.); 3 Natural History Museum of Denmark, University of Copenhagen, Universitetsparken 15, DK-2100 Copenhagen Ø, Denmark; aneelises@snm.ku.dk; 4 Fossil and Moclay Museum, Museum Mors, Skarrehagevej 8, DK-7900 Nykøbing Mors, Denmark; 5 Agilent Technologies Denmark ApS, Produktionsvej 42, DK-2600 Glostrup, Denmark; ronjamathiesen@gmail.com

**Keywords:** animal behaviour, applied conservation biology, *Erinaceus europaeus*, human–wildlife conflicts, robotic lawn mowers, wildlife conservation

## Abstract

**Simple Summary:**

Injured European hedgehogs are frequently admitted to hedgehog rehabilitation centres with different types of cuts and injuries. Although not rigorously quantified, a growing concern is that an increasing number of cases may have been caused by robotic lawn mowers. Research indicates that European hedgehogs are in decline. It is therefore important to identify and investigate the factors responsible for this decline to improve the conservation initiatives directed at this species. Because hedgehogs are increasingly associated with human habitation, it seems likely that numerous individuals will encounter several robotic lawn mowers during their lifetimes. Consequently, this study aimed to describe and quantify the effects of robotic lawn mowers on hedgehogs, and we tested 18 robotic lawn mowers in collision with dead hedgehogs. Some models caused extensive damage to the dead hedgehogs, but there were noteworthy differences in the degree of harm inflicted, with some consistently causing no damage. None of the robotic lawn mowers tested was able to detect the presence of dead, dependent juvenile hedgehogs, and no models could detect the hedgehog cadavers without physical interaction. We therefore encourage future collaboration with the manufacturers of robotic lawn mowers to improve the safety for hedgehogs and other garden wildlife species.

**Abstract:**

We tested the effects of 18 models of robotic lawn mowers in collision with dead European hedgehogs and quantified the results into six damage categories. All models were tested on four weight classes of hedgehogs, each placed in three different positions. None of the robotic lawn mowers tested was able to detect the presence of dependent juvenile hedgehogs (<200 g) and all models had to touch the hedgehogs to detect them. Some models caused extensive damage to the hedgehog cadavers, but there were noteworthy differences in the degree of harm inflicted, with some consistently causing no damage. Our results showed that the following technical features significantly increased the safety index of the robotic lawn mowers: pivoting blades, skid plates, and front wheel drive. Based on these findings, we encourage future collaboration with the manufacturers of robotic lawn mowers to improve the safety for hedgehogs and other garden wildlife species.

## 1. Introduction

Research on both national and local scales has either documented, or expressed concern about the likelihood of, a decline in European hedgehog (*Erinaceus europaeus*) populations in several western European countries [1,2,3,4,5,6,7,8,9,10]. It is therefore a priority to identify and investigate the factors responsible for this decline to provide the evidence necessary to underpin remedial conservation interventions.

Injured hedgehogs are frequently admitted to hedgehog rehabilitation centres with different types of cuts and injuries. Some injuries are consistent with known risks to hedgehogs in the form of garden trimmers and dog bites [11,12,13]. However, although not rigorously quantified, a concern has arisen in several European countries that an increasing number of cases may have been caused by robotic lawn mowers. Although not previously investigated, these growing rumours have led to several articles in the media and on social media claiming that these mowers are lethal to hedgehogs. If the threat is real, then it would indeed be a cause for concern, as the global market for robotic lawn mowers is expanding dramatically and was expected to reach USD 1.3 billion in 2020, growing at an annual rate of more than 12 percent during the period 2019–2025 [14].

As research indicates that European hedgehogs are increasingly associated with human habitation [7,8,15,16,17] and are often seen foraging on grassy turf in the gardens and green spaces of urban areas [18,19,20,21,22], it seems likely that numerous individuals will encounter several robotic lawn mowers during their lifetimes. To our knowledge, there has thus far been no systematic scientific research evaluating whether this risk of physical damage is mere hearsay or a real and present threat to be added to the already vulnerable species. Therefore, the aims of this study are to describe and quantify the physical effects of robotic lawn mowers on hedgehogs and provide information on potential technical features of the machines that could increase the safety index of the robotic lawn mowers. The main purpose of providing this information is to improve the conservation of European hedgehogs living in residential areas by reducing the plausible negative anthropogenic effects potentially caused by robotic lawn mowers.

## 2. Materials and Methods

In total, 18 designs of robotic lawn mowers were selected for the study. The selection was based on the advice of a product specialist in robotic lawn mowers and is considered to represent the spectrum of brands, models, and specifications of the products available on the European market (Table 1). The cutting height of the machines was adjusted to the highest setting to keep the grass at the test site intact to ensure equal test conditions for all trials.

**Figure 1:**
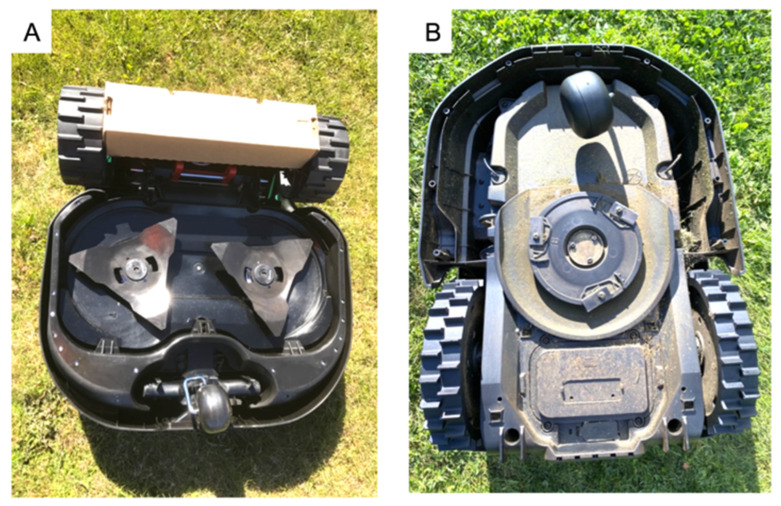
Fixed or pivoting blades. (**A**) A robotic lawn mower with fixed blades. (**B**) A robotic lawn mower with pivoting blades. Pivoting blades will fold into a protective frame when hitting something harder than grass, as opposed to fixed blades, which are constantly exposed. Photographs by Petrus Ekbladh and Ronja Mathiesen.

Of the 18 robotic lawn mowers tested, 5 had fixed blades (Figure 1A) and 13 had pivoting blades (Figure 1B).

The robotic lawn mower tests were performed on dead hedgehogs, henceforth referred to as “hedgehogs”. These animals had died in, and were secured from, hedgehog rehabilitation centres in Denmark from June to August 2020. All hedgehogs chosen for this study were intact with no visible injuries. The hedgehogs were stored in freezers at −20 °C and were thawed before the tests. The 70 selected hedgehog cadavers were divided into four different weight classes to represent four stages of life (Table 2).

Each robotic lawn mower model was tested on four hedgehogs representing each of the four described weight classes. If an individual was injured by the mower during a test, it would be discarded to avoid confusion or interaction with previous injuries in subsequent tests (with one uncomplicated exception, where we reused a cadaver with superficial injuries in weight class 4, due to a shortage of individuals in this category).

**Table 2:** Weight classes. Graphical representation of the four weight classes of dead hedgehogs used in the study. The pictures of live hedgehogs were provided to illustrate the sizes of individuals belonging to the four weight classes. No live hedgehogs were tested in this study. The ruler on the pictures indicates length of the individuals in cm. Photographs by Michela Dugar.

Weight Class	Weight (g)	No. of Individuals	Total No. of Individuals per Weight Class	Stages of Life	Representation	
1	Up to 199	22	22	Dependent juveniles	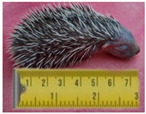	Weight 46 g Length 7.5 cm
2	200	3	21	Independent juveniles	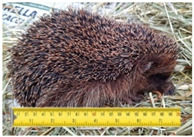	Weight 530 g Length 19.5 cm
	300	3				
	400	9				
	500	6				
3	600	8	20	Adults	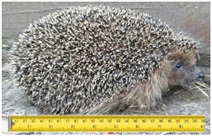	Weight 860 g Length 23 cm
	700	8				
	800	4				
4	900	4	7	Large adults	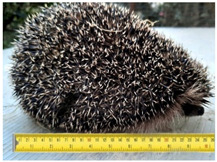	Weight 1080 g Length 25 cm
	1000	2				
	1100	1				

**Figure 2:**
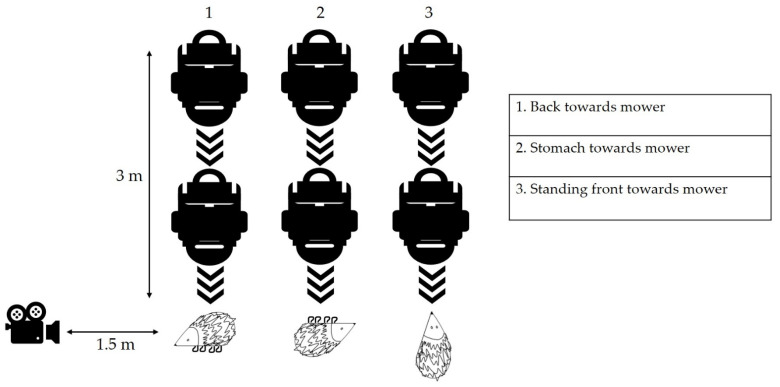
An overview of the test setup. Each robotic lawn mower was tested 12 times in total, 3 times per dead hedgehog representing 1 of 4 weight classes. A hedgehog from each of the four weight classes was placed in three different positions. The three positions were (**1**) Lying on the side with the back oriented towards the approaching robotic lawn mower; (**2**) Lying on the side with the stomach oriented towards the approaching robotic lawn mower; (**3**) Standing upright on its feet with the head oriented towards the approaching robotic lawn mower. The damage recorded from each test was categorised as 0-A according to the damage categories.

Each individual was tested in three different positions (Figure 2), as an attempt to mimic the behaviour of a live individual:

1. Lying on the side with the back pointing towards the approaching robotic lawn mower, mimicking the curled up position a hedgehog often adopts as a defence mechanism against approaching danger [19,23].
2. Lying on the side with the stomach pointing towards the approaching robotic lawn mower (somewhat unnatural, but extremely vulnerable position).
3. Standing upright on its feet with the head pointing towards the approaching robotic lawn mower (an expression of curiosity but not alarm).

To sum up, each robotic lawn mower was tested 12 times:

- Three times per individual (once in each of the three positions).
- One individual from each of the four weight classes.

The tests were filmed with a GoPro Hero 8 Black action camera placed on a tripod. If a hedgehog was injured during the tests, we recorded the injuries and documented them with the camera.

The tests of 17 out of 18 machines were carried out in a private garden in Hok, Sweden, with a flat and well-trimmed lawn, on 25 and 26 August 2020. The last machine (model: Grouw M900) was tested in a private garden with a flat and well-trimmed lawn in Aarhus, Denmark on 25 September 2020. All 216 tests were performed during daylight.

The setup for most of the tests was as follows (Figure 2): The hedgehog was placed on the lawn at a 3 m distance from the robotic lawn mower. The camera was placed next to the hedgehog on the left-hand side at a 1.5 m distance. The mower was then turned on and manually directed to move towards the hedgehog. The distance of 3 m was sufficient to ensure the machine was operating at maximum speed, and the blades were in action, before reaching the hedgehog. If the machine did not move in a straight line towards the hedgehog, it was then relocated back to the initial position and turned on again. This was done to standardise the tests and to ensure that the hedgehog was located to the centre of the front of each approaching machine.

In the cases of two models (tests on Stiga Autoclip 530 SG (Stiga, Castelfranco Venetto, Iltaly) and Ambrogio Robot 4.0 Elite (Zucchetti Centro Sistemi Spa, Arezzo, Italy)) the machine was turned on at a greater distance than 3 m from the hedgehog cadaver, because these particular robotic lawn mowers took longer distances to gain momentum and for their blades to be functioning fully. Due to its specifications (causing erratic movements), the tests of the model Honda Miimo HRM 40 Live (Honda France Manufacturing, Ormes, France) were filmed with a mobile camera, with the hedgehog placed in front of the approaching machine once it had gained full speed.

### 2.1. Quantifying the Damage 

We divided severity of damage caused by the robotic lawn mowers into six damage categories:

0. No physical contact between the machine and the hedgehog. The machine senses the hedgehog from a distance, changes direction, and drives on without touching the hedgehog. No damage is caused to the hedgehog cadaver.
1. The robotic lawn mower approaches the hedgehog and the front of the machine touches the hedgehog lightly (a “nudge”) and thereby detects the corpse. Immediately, the machine changes direction and drives on without touching the hedgehog further. No damage is caused to the hedgehog cadaver.
2. The robotic lawn mower approaches the hedgehog and the front of the machine touches the hedgehog (a “flip”) to detect the hedgehog. The physical interaction causes the hedgehog to be moved into a different body position (flipped from lying on one side of the body to the other side of the body) or being lifted partly from the ground before settling in the same position again. Afterwards, the machine changes direction and drives on without touching the hedgehog further. The damage to the hedgehog is at most minimal and involves no contact with the blades (at worst this might cause a slight bruise).
3. The robotic lawn mower fails to detect the presence of the hedgehog and continues to drive across the hedgehog. The front panel of the machine is lifted as the machine drives over the cadaver, which causes the blades to stop running [24]. In some cases the machine withdraws and changes direction, so that only part of the dead hedgehog’s body was situated underneath the machine. The blades of the robotic lawn mower may have come into contact with the dead hedgehog but have not punctured the skin. The damages observed ranged from undetectable to the cutting of a small number of spines, but might have involved minor bruising to a live hedgehog.
4. The robotic lawn mower fails to detect the presence of the hedgehog and continues to drive across it. The blades of the machine have come into contact with the dead hedgehog and have caused injuries to the cadaver. The severity of the injuries range from small puncture wounds on the skin (1 cm) to clipping of limbs or complete exposure of the entire abdominal region and decapitation.
A. The machine does not detect the juvenile hedgehog (<200 g, weight class 1) and continues to drive across it. As the body of the small hedgehog is situated below the blades of the robotic lawn mower, the juvenile hedgehog is left with no visible injuries. It is possible that in life this could have caused injury or bruising, perhaps by the wheels rather than the blades (and much would depend on the response of the juvenile hedgehog in life).

### 2.2. Data Analyses

The proportion (ratio) of “no damage” or “damage” during the tests, the safety index, was calculated for the following features on the mowers: (1) blade type, (2) front or rear wheel drive, (3) wheel numbers, (4) skid plate, (5) ultrasonic sensor, (6) camera vison, (7) collision sensors, and (8) wheel motor current collision detection.

For the data analyses, the damage categories were divided into two definitions of “no damage” and “damage”:

- “No damage”:
  1. Pooled damage categories 0, 1, and 2.
  2. Pooled damage categories 0, 1, 2, and 3.
- “Damage”:
  1. Pooled damage categories 3 and 4.
  2. Only damage category 4.

The ratios of “no damage”/”damage” were interpreted as an overall index of safety for the hedgehogs. The higher the number of “no damage” events compared to “damage”, the higher the safety index, and hence the judgement that the robotic lawn mower was more "hedgehog friendly".

The statistical significance for each of the “no damage”/”damage” ratios was tested with 2 × 2 Chi square tests with Yate’s correction (Chi_Yate’s correction_) to investigate if the presence or absence of a given technical feature on a robotic lawn mower significantly affected the ratios.

The 2 × 2 Chi square tests with Yate’s correction were firstly conducted for each of the four weight classes and three positions of the hedgehogs, separately. However, due to the low statistical power caused by analysing weight and position separately, all weight classes and positions were combined. Subsequently, 2 × 2 Chi square tests with Yate’s correction for all weight classes and positions combined were calculated for each of the two definitions of damage, testing the effect of each of the eight chosen technical features on the robotic lawn mowers on the safety index.

Lastly, we calculated the percentage distribution of damage to hedgehogs during the 12 tests on each mower based on the total number of cases where damage was recorded (either damage category 3 + 4 or damage category 4). Damage category A was omitted from the analyses, because including it resulted in different sample sizes of tests for different models of robotic lawn mowers. Therefore, the percentage distribution was chosen as a measure of safety.

## 3. Results

Regardless of brand, model, and specifications, none of the robotic lawn mowers detected the dependent juvenile hedgehogs (<200 g, weight class 1). Some machines did, however, move over the individuals resulting in no apparent damage, as the juveniles were sufficiently small, i.e., smaller than the minimum mowing height, thereby avoiding the running blades of the mowers (damage category A).

**Figure 3:**
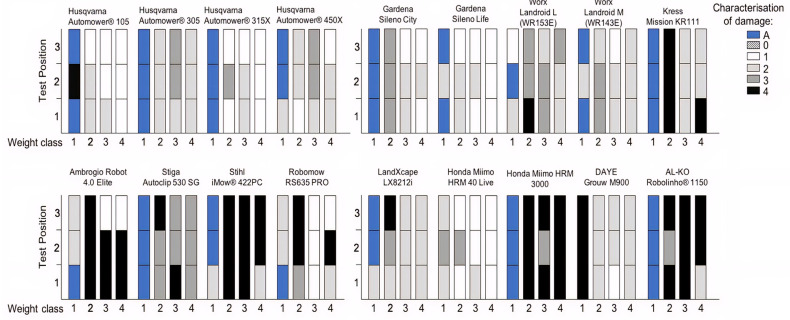
The test results for each of the 18 robotic lawn mowers tested. Every result for each of the four weight classes in each of the three positions have been described based on a categorisation of damage ranging from 0 to 4, with damage category 4 being the most severe. Damage category A represents the events where the machine does not detect the juvenile hedgehog (<200 g, weight class 1) and continues to drive across the juvenile hedgehog, but as the body of the small hedgehog is situated below the blades of the robotic lawn mower, the juvenile hedgehog is left with no visible injuries or bruises.

In all tests of weight category classes > 200 g (weight classes 2–4), the robotic lawn mowers had to physically interact with the hedgehog cadaver to detect it. None of the machines, not even models with camera vision and ultrasonic sensors, was able to detect the hedgehog in advance and change direction before touching the hedgehog. Therefore, we did not record any damage category 0. In many cases, the mowers would only touch the hedgehog (damage category 1 or 2), subsequently detect it, and change direction. However, some machines did not detect the hedgehogs and ran straight over them. In some cases the mandatory safety measures of the machine [24] caused the blades to stop rotating within seconds of contact, leaving the hedgehog undamaged or with slight cuts to the spines (damage category 3). In the event that the safety features of the machine failed to detect the hedgehog, the result was injury to the cadaver (damage category 4) ranging from lighter skin abrasions and puncture wounds, to the amputation of extremities like legs and penises, to complete disembowelment, and in one case a partial decapitation. The injuries appeared on all areas of the body in no particular pattern, as it depended on the position in which the hedgehog was caught under the robotic lawn mower, as well as the angles of the blades. Figure 3 provides an overview of the damage categories recorded for each of the 12 different tests performed on the 18 robotic lawn mowers.

Comparing the effect of fixed and pivoting blades, the results showed that pivoting blades significantly reduced the number of damages during the tests, regardless of the definition of the category “damage” (either damage category 3 + 4 or damage category 4) (Chi_Yate’s correction_ = 28.95 and 26.62, *p* < 0.0001). The same applied to machines with front wheel drive compared to rear wheel drive (Chi_Yate’s correction_ (4) = 7.25, *p* = 0.007; Chi_Yate’s correction_ (3 + 4) = 8.99, *p* = 0.003) as well as the presence of skid plates on the machines (Chi_Yate’s correction_ = 11.39 and 10.99, *p* = 0.001). Robotic lawn mowers with three wheels instead of four had a significantly higher safety index, meaning that there were fewer cases of damage to the hedgehogs during the tests for the damage categorisation based on both damage category 3 and 4 (Chi_Yate’s correction_ = 4.37, *p* = 0.037), but not for the damage categorisation based only on damage category 4. Ultrasonic sensors also appeared to increase the safety index for the damage categorisation based on damage category 3 and 4 (Chi_Yate’s correction_ = 3.84, *p* = 0.05), but not for the damage categorisation based only on damage category 4. The presence of collision sensors, compared to wheel motor current collision detection, reduced the safety index for damage category 4 (Chi_Yate’s correction_ = 13.23, *p* = 0.0003). Table 3 provides a summary of the Chi square statistics.

The percentage distribution of damages to the hedgehogs during the tests of each robotic lawn mower (Table 4) provides an overview of the performance of each machine. The lower percentage of damages during the tests, the safer the mower is for hedgehogs, insofar as the injuries are a good approximation to what could be sustained on live hedgehogs. The percentage distribution of damages varied accordingly, as some models may have caused no or few category 4 damages but had a higher occurrence of damage category 3 during the tests.

## 4. Discussion

As the results showed that none of the robotic lawn mowers tested was able to detect the hedgehogs without physical interaction and none detected the dependent juveniles, we cannot be confident that any of the robotic lawn mowers tested were entirely safe to hedgehogs. Preferably, the machines should not interact physically at all with the hedgehogs. However, the damages categorised as 1–2 do not appear to harm the hedgehogs, and perhaps the hedgehogs may even learn to avoid robotic lawn mowers after such an encounter. Furthermore, there were obvious differences in the outcome on the hedgehogs depending on the machines tested, with some models consistently causing no damage on collision (Figure 3 and Table 4).

Some of the injuries recorded would have been immediately lethal, and all of the damages in category 4 would have had the potential to become lethal if left untreated. A small puncture wound, if untreated, might get infected and progress to balloon syndrome, a potentially lethal condition caused by subcutaneous emphysema, which makes the skin of the hedgehog blow up like a balloon [25], or a general systemic infection. As hedgehogs are considered quite elusive even when damaged and in pain, it must be assumed that a proportion of hedgehogs injured by robotic lawn mowers will not be found and helped in time and will likely die from their injuries in the wild.

In some cases the robotic lawn mowers failed to detect the hedgehog but met the safety regulations insofar as the blades stopped when the surface of the machine was lifted (activating a tilt-, lift- or obstruction sensor) leaving the skin of the hedgehog unbroken (damage category 3) [24]. However, there were situations where the mower continued to run over and hence injure the hedgehog cadaver. We reduced our recording of injuries to one category (damage category 4), as the outcome may be influenced by a range of different factors, such as the soil softness and type, height of the grass, position of the hedgehog as the robotic lawn mower runs over the individual, and how the collision with the hedgehog positions the individual underneath the blades. As these different factors may have influenced the results of the tests, causing uncertainty of the potential outcome in all scenarios where the robotic lawn mowers failed to detect the hedgehogs and continued to run over the individuals (damage categories 3 and 4), we decided to represent both types of damage categories in our analyses of the results (damage category 4 and damage category 3 + 4) as a precautionary measure.

During our experiments, there was a greater likelihood that robotic lawn mowers with fixed blades would fail to detect the dead hedgehogs, causing more extensive damage to them. These results may be explained by various factors. In contrast to fixed blades, which are constantly exposed, pivoting blades fold into a protective frame when they hit something harder than grass. Furthermore, robotic lawn mowers with fixed blades require more heavy-duty machine power to run the blades, and this greater power appeared to render the machines less controllable and less sensitive in their detection technology. The engineering of front- compared to rear-wheel drive, as well as the use of three compared to four wheels, influenced the safety index positively. This may also be because models with front-wheel drive and three wheels all had pivoting blades. The same explanation may apply to the significantly lower incidence of damage for tests on robotic lawn mowers with ultrasonic sensors, all of which were fitted with pivoting blades. Lastly, the presence of skid plates significantly reduced the number of tests causing damage to the hedgehogs. The skid plate is designed to protect the pivoting blades from hard objects and thereby also protects foreign objects, such as a hedgehog, from the blades. Only one of the models tested contained a combination of these beneficial features (except ultrasonic sensors). These should be the focus of future designs of robotic lawn mowers with hedgehog safety in mind.

We cannot rule out the possibility that the results were also influenced by the lift detection sensitivity of the robotic mowers. We could not test this, but presume that if lift detection sensitivity was sufficiently high, the machines would detect the hedgehogs and change direction or stop the blades rotating as soon as the surface of the machine was lifted, reducing the risk of injuries.

### 4.1. Using Dead Hedgehogs as Test Subjects

Working with dead hedgehogs as test subjects may not perfectly mimic the outcomes of natural collisions. Firstly, live hedgehogs might detect and evade the robotic lawn mower. Secondly, they might curl up, and their tightened muscles and raised spines could provide protection. We sought to mimic these behaviours in the positions we chose for the cadavers, but of course their muscle tone and reactions were different. Alternative insights would come from simulations using live hedgehogs with safely modified mowers.

### 4.2. Failed Detection of Dependent Juveniles and the Consequences

None of the tested robotic lawn mowers was able to detect the dependent juvenile hedgehogs (<200 g, weight class 1). In most cases, these small individuals passed beneath the rotating blades. We do not know how mother hedgehogs accompanied by their litters would react to an active robotic lawn mower, but reports from the public indicate that they generally tend to stay in the nests during ordinary human garden activity, although this would have to be investigated further in future work. An orphaned juvenile hedgehog is more likely to be exposed to running robotic lawn mowers. However, such an individual is already very vulnerable with a low chance of survival, regardless of the presence of a robotic lawn mower, unless found in good time and taken into care at a wildlife rehabilitation centre.

### 4.3. Results in Relation to Discussions with the Public

The public discourse has raised questions of whether hedgehogs can outrun robotic lawn mowers and whether hedgehogs are able to detect them properly. As we used cadavers, we were not able to test this, but we do know that hedgehogs can run at up to 50 m per minute [26], whereas the maximum speed of Husvarna’s robotic lawn mowers ranges between 21 m per minute and 39 m per minute (pers. comm. Husqvarna). In terms of cues likely to alert the hedgehogs, these machines make characteristic sounds and smells detectable by human senses. We made no observations of the behavioural responses of live hedgehogs to the mowers, although this could be done at no risk to the hedgehogs using disarmed machines.

As hedgehogs are nocturnal, it has been widely recommended that any problem would be circumvented by running robotic mowers only by day. This might indeed largely obviate the problem, nonetheless being mindful that hedgehogs may be active during the daytime for several different reasons [19,23].

In the light of the results from the present study, we encourage manufacturers, distributors, and sellers of robotic lawn mowers to educate customers on the importance of refraining from using robotic lawn mowers at night time and to check the lawn for wildlife species that are potentially vulnerable to the machines, such as hedgehogs, leverets, fledglings, and amphibians, before mowing.

## 5. Conclusions

As hedgehogs are increasingly associated with human habitation, they are likely to encounter robotic lawn mowers, and our results show the encounters, depending on the model, could be injurious and even fatal. That said, while our study answers critical questions regarding the likely nature and extent of injuries, we cannot comment on the likelihood of these encounters or the hedgehogs’ responses to them. However, a major step towards resolving the risk of robotic lawn mowers on hedgehog survival involves the design and purchase of hedgehog-friendly mowers, a topic of potentially fruitful collaboration between hedgehog conservationists, behavioural ecologists, and mower manufacturers.

## Figures and Tables

**Table 1:** Overview of the models of robotic lawn mowers tested. In the column “Blades”, Pivoting indicates “low energy pivoting blades” and Fixed indicates “heavy duty fixed blades”. WMCC detection is short for “wheel motor current collision detection”.

Test Number	Brand	Model	Blades	Collision Sensor	WMCC Detection	Wheels	Front/Rear Wheel Drive	Skid Plate	Headlights	Ultrasonic Sensors	Camera Vision
1	Husqvarna	Automower^®^ 105	Pivoting	Yes		3	Front	Yes			
2	Husqvarna	Automower^®^ 305	Pivoting		Yes	4	Rear	Yes			
3	Husqvarna	Automower^®^ 315X	Pivoting	Yes		4	Rear	Yes	Yes		
4	Husqvarna	Automower^®^ 450X	Pivoting	Yes		4	Rear	Yes	Yes	Yes	
5	Gardena	Sileno City	Pivoting		Yes	3	Front				
6	Gardena	Sileno Life	Pivoting		Yes	4	Front				
7	Worx	Landroid L (WR153E)	Pivoting		Yes	4	Rear				
8	Worx	Landroid M (WR143E)	Pivoting		Yes	4	Rear			Yes	
9	Kress	Mission KR111	Pivoting	Yes		4	Rear			Yes	
10	LandXcape	LX8212i	Pivoting		Yes	3	Rear			Yes	
11	Honda	Miimo HRM 40 Live	Pivoting		Yes	4	Rear				
12	Honda	Miimo HRM 3000	Pivoting	Yes		4	Rear				
13	Robomow	RS635 PRO	Fixed		Yes	3	Rear				Yes
14	AL-KO	Robolinho^®^ 1150	Fixed		Yes	4	Rear				
15	Ambrogio Robot	4.0 Elite	Fixed	Yes		4	Rear				
16	Stiga	Autoclip 530 SG	Fixed		Yes	4	Rear				
17	Stihl	iMow^®^ 422PC	Fixed	Yes		4	Rear				
18	DAYE	Grouw M900	Pivoting	Yes		4	Rear				

**Table 3:** Results from the data analyses investigating if the presence or absence of a given technical feature on a robotic lawn mower significantly influenced the safety index. Damage category A was omitted from the analyses.

Features	Damage Categories Included	Type	No Damage	Damage	Safety Index (No Damage/Damage)	Safety Index	Chi Square with Yates Correction	*p*-Value
Fixed or pivoting blades	4	Fixed	23	27	23/27	**0.85**	28.95	<0.0001 ***
		Pivoting	112	18	112/18	**6.22**		
	3 + 4	Fixed	14	36	14/36	**0.39**	26.62	<0.0001 ***
		Pivoting	93	37	93/37	**2.51**		
Front or rear wheel drive	4	Front	28	1	28/1	**28.00**	7.25	0.007 **
		Rear	107	44	107/44	**2.43**		
	3 + 4	Front	25	4	25/4	**6.25**	8.99	0.003 **
		Rear	82	69	82/69	**1.19**		
3 or 4 wheels	4	3 wheels	35	5	35/5	**7.00**	3.47	**0.062**
		4 wheels	100	40	100/40	**2.50**		
	3 + 4	3 wheels	30	10	30/10	**3.00**	4.37	0.037 *
		4 wheels	77	63	72/63	**1.22**		
Skid plate	4	Yes	37	1	37/1	**37.00**	11.39	0.001 ***
		No	98	44	98/44	**2.23**		
	3 + 4	Yes	32	6	32/6	**5.33**	10.99	0.001 ***
		No	75	67	75/67	**1.12**		
Ultrasonic sensors	4	Yes	34	5	34/5	**6.80**	3.15	**0.076**
		No	101	40	101/40	**2.53**		
	3 + 4	Yes	29	10	29/10	**2.90**	3.84	0.050 *
		No	78	63	78/63	**1.24**		
Camera vision	4	Yes	8	3	8/3	**2.67**	0.03	**0.857**
		No	127	42	127/47	**3.02**		
	3 + 4	Yes	7	4	7/4	**1.75**	0.01	**0.980**
		No	100	69	100/69	**1.45**		
Collision sensors	4	Yes	49	31	49/31	**1.58**	13.23	0.0003 ***
		No	86	14	86/14	**6.14**		
	3 + 4	Yes	45	35	45/35	**1.29**	0.39	**0.53**
		No	62	38	62/38	**1.63**		
Wheel motor current collision detection	4	Yes	86	14	86/14	**6.14**	13.23	0.0003 ***
		No	49	31	49/31	**1.58**		
	3 + 4	Yes	62	38	62/38	**1.63**	0.39	**0.53**
		No	45	35	45/35	**1.29**		

* *p*-value ≤ 0.05, ** *p*-value ≤ 0.01, *** *p*-value ≤ 0.001.

**Table 4:** The percentage distribution of tests resulting in damage to the hedgehogs, defined either as damage category 4 or damage category 3 + 4. Damage category A was omitted from the analyses, leaving the total number of tests between 9 and 12 depending on the amount of damage category A results recorded per robotic lawn mower. The lower percentage of cases of damage during the tests, the safer the robotic lawn mower. The robotic lawn mower models have been listed in accordance with the percentage distribution of damage defined as damage category 3 + 4. Models showing the lowest damage percentage are listed first.

Robotic Lawn Mowers		Tests with Damage Category 4			Tests with Damage Category 3 + 4		
Brand	Model	No Damage (0–3)	Damage (4)	Cases of Damage in Tests (%)	No Damage (0–2)	Damage (3–4)	Cases of Damage in Tests (%)
Gardena	Sileno Life	10	0	**0**	10	0	**0**
Husqvarna	Automower^®^ 105	9	1	**10**	9	1	**10**
Husqvarna	Automower^®^ 315X	9	0	**0**	8	1	**11**
Honda	Miimo HRM 40 Live	12	0	**0**	10	2	**17**
Husqvarna	Automower^®^ 450X	10	0	**0**	8	2	**20**
Worx	Landroid M (WR143E)	10	0	**0**	8	2	**20**
LandXcape	LX8212i	9	1	**10**	8	2	**20**
Husqvarna	Automower^®^ 305	9	0	**0**	7	2	**22**
DAYE	Grouw M900	9	3	**25**	9	3	**25**
Gardena	Sileno City	9	0	**0**	6	3	**33**
Robomow	RS635 PRO	8	3	**27**	7	4	**36**
Kress	Mission KR111	5	4	**44**	5	4	**44**
Worx	Landroid L (WR153E)	10	1	**9**	5	6	**55**
Ambrogio Robot	4.0 Elite	4	7	**64**	4	7	**64**
Stihl	iMow^®^ 422PC	2	8	**80**	2	8	**80**
AL-KO	Robolinho^®^ 1150	2	7	**78**	1	8	**89**
Honda	Miimo HRM 3000	1	8	**89**	0	9	**100**
Stiga	Autoclip 530 SG	7	2	**22**	0	9	**100**

## Data Availability

Further data from the research is available from Zenodo (DOI: 10.5281/zenodo.4707658, https://doi.org/10.5281/zenodo.4707658).
